# Comparison of Three Different Multiple Organ Dysfunction Scores for Predicting Mortality after Neonatal Cardiac Surgery

**DOI:** 10.3390/children10081333

**Published:** 2023-08-01

**Authors:** Handan Bezirganoglu, Nilufer Okur, Fatih Ozdemir, Ozlem Gul, Bedri Aldudak

**Affiliations:** 1 Division of Neonatology, Trabzon Kanuni Training and Research Hospital, Trabzon 61250, Türkiye; 2 Division of Neonatology, Diyarbakir Gazi Yasargil Training and Research Hospital, Diyarbakir 21010, Türkiye; 3 Department of Pediatric Cardiovascular Surgery, Dr. Siyami Ersek Thoracic and Cardiovascular Surgery Training and Research Hospital, Istanbul 34668, Türkiye; 4 Division of Pediatric Cardiology, Diyarbakir Gazi Yasargil Training and Research Hospital, Diyarbakir 21010, Türkiye

**Keywords:** congenital heart disease, neonatal multiorgan dysfunction score, mortality, cardiac surgery

## Abstract

Infants who undergo cardiac surgery frequently have complications that may advance to multiple organ failure and result in mortality. This study aims to compare three different multiple organ dysfunction scoring systems: the Neonatal Multiple Organ Dysfunction (NEOMOD) score, the modified NEOMOD score, and the Pediatric Logistic Organ Dysfunction-2 (PELOD-2) score in predicting postoperative 30-day mortality in neonates undergoing cardiac surgery. This retrospective cohort study was conducted between January 2019 and February 2021 in a single unit on neonates operated on due to congenital heart disease in the first 28 days of life. Patients who underwent off-pump surgeries were excluded from the study. The NEOMOD, modified NEOMOD, and PELOD-2 scores were calculated for each of the first 3 days following surgery. A total of 138 patients were included. All scores had satisfactory goodness-of-fit and at least good discriminative ability on each day. The modified NEOMOD score consistently demonstrated the best prediction among these three scores after the first day, reaching its peak performance on day 2 (area under curve: 0.824, CI: 0.75–0.89). Our findings suggest that NEOMOD and modified NEOMOD scores in the first 72 h could potentially serve as a predictor of mortality in this population.

## 1. Introduction

The survival rate of neonates diagnosed with congenital heart disease (CHD) has shown a substantial improvement in conjunction with advancements in medical and surgical management, as well as post-operative care [1]. However, infants undergoing cardiac surgery still face the risk of developing complications that can potentially lead to multiple organ failure and mortality. Hence, it is crucial to use reliable indices to objectively assess the risk of death and prognosis following cardiac surgery in order to facilitate better clinical care and decision making regarding treatment options.

Multiple organ dysfunction syndrome (MODS) may emerge after cardiac surgery as a result of the cumulative effect of cardiopulmonary bypass and the surgical trauma, leading cardiovascular instability, endothelial damage, and activation of complex humoral and cellular-mediated immune responses [2]. In pediatric cardiac patients, a study by Ben-Abraham et al. revealed that MODS accounted for 81.8% of mortality in the first week following cardiac surgery [3]. Furthermore, studies have also shown that the incidence of MODS and mortality rate were significantly higher in neonates than in older children after cardiac surgery [4,5].

The Pediatric Logistic Organ Dysfunction-2 score (PELOD-2) has been validated to be used as an objective measure of organ dysfunction in critically ill children [6]. Although the PELOD-2 score has good discriminative values and is used as an alternative outcome in pediatric studies for risk adjustment, there is a scarcity of data regarding its application in pediatric cardiac surgery [7,8]. In neonates with MODS, the Neonatal Organ Dysfunction score (NEOMOD) is commonly used to predict mortality in critically ill premature infants [9]. Moreover, the modified NEOMOD score is developed by including involvement of the microvascular system for early detection of MODS in infants [10]. However, neither of these two organ dysfunction scores has been used in neonates with CHD.

The commonly used risk and/or complexity scores, Risk Adjustment for Congenital Heart Surgery (RACHS-1) and Aristotle Basic Complexity (ABC), have been found to have low predictive value for mortality in the neonatal population [11]. Additionally, these scores demonstrate weak correlation with the PELOD score in pediatric cardiac patients [12]. As a result, the development of appropriate organ dysfunction scores for predicting worse outcomes in neonatal postoperative cardiac surgery is needed.

Thus, in this study, we aimed to evaluate the usefulness of the PELOD-2, NEOMOD, and modified NEOMOD scores in predicting mortality in neonates following cardiac surgery.

## 2. Materials and Methods

### 2.1. Study Population

This retrospective cohort study was conducted between January 2019 and February 2021 in a single Neonatal Intensive Care unit (NICU) at the Gazi Yaşargil Training and Research Hospital, Diyarbakır, Turkey. The study was conducted in accordance with the Declaration of Helsinki after approval from the ethics committee of our hospital (121;24.06.2022). All infants who underwent cardiac surgery due to congenital heart disease in the first 28 days of life were eligible for inclusion. Infants with congenital or chromosomal anomalies, who were operated on without cardiopulmonary bypass (CPB), died within the first 12 h after surgery, and whose data were missing were excluded from the study.

### 2.2. Demographic and Clinical Data

Demographic and clinical data were obtained by abstracting information from electronic records and patients’ files. Preoperative clinical characteristics, including gestational age, gender, birth weight, prematurity, small for gestational age (SGA), age at surgery, weight at surgery, underlying cardiovascular diagnosis, preoperative organ failure and need for preoperative mechanical ventilation were obtained from medical records. Cardiopulmonary bypass was conducted following standardized protocols by the same perfusion team. Perioperative data, including RACHS-1 scores, duration of cardiopulmonary bypass, duration of aortic cross clamping, and need for delayed sternal closure were also collected. Postoperative complications, vasoactive inotropic score (VIS) at day 1 and day 2, neonatal intensive care length of stay, and hospital length of stay were recorded. VIS values were calculated as follows: dopamine dose (µg/kg/min) + dobutamine dose (µg/kg/min) + 100 × epinephrine dose (µg/kg/min)] + 10 × milrinone dose (µg/kg/min) + 10,000 × vasopressin dose (units/kg/min) + 100 × norepinephrine dose (µg/kg/min). Mortality was defined as the patient dying after surgery but before discharge from hospital, or death after hospital discharge but within 30 postoperative days.

### 2.3. Organ Dysfunction Scores

The assessment of organ dysfunction was performed using three scoring systems: the PELOD-2, the NEOMOD, and the modified NEOMOD scores. Organ dysfunction scores were calculated at three postoperative time intervals: within the initial 12–24 h, on day 2, and on day 3 after NICU admission.

The PELOD-2 score evaluates five organ functions using ten parameters: neurologic (Glasgow coma score and pupillary reaction), cardiovascular (lactatemia, mean arterial pressure), renal (creatinine), respiratory (PaO_2_/FiO_2_ ratio, PaCO_2_, invasive ventilation), and hematologic (white blood cell count and platelets) [13]. Each parameter is assigned a score ranging from 0 to 6, and the total PELOD-2 score is calculated as the sum of these individual scores. The highest possible score is 33, and the minimum is 0. For the NEOMOD score and the modified NEOMOD score, organ dysfunction in each system was determined based on laboratory tests and clinical evaluation. The NEOMOD system assesses dysfunction in seven organ systems: neurologic, cardiovascular, renal, respiratory, gastrointestinal, hemocoagulation balance, and acid–base balance, whereas the modified NEOMOD includes the microvascular system (albumin, degree of edema) as an additional system [9,10]. Furthermore, the modified NEOMOD score includes additional variables: white blood cell counts, creatinine, alanine transaminase, total bilirubin, and albumin levels. In both scoring systems, each organ system is classified as normal (0 points), moderate dysfunction (1 point), or severe dysfunction (2 points). The maximum possible score is 14 points for the NEOMOD score and 16 points for the modified NEOMOD score.

For PELOD-2 score, the most abnormal value of each variable observed during the specific time intervals was considered for calculation. For NEOMOD and modified NEOMOD scores, criteria of feeding for the gastrointestinal systems were defined as follows: a score of ‘2’ was assigned if there was a sign of necrotizing enterocolitis, a score of ‘1’ if there was a need for total parenteral nutrition, and a score of ‘0’ if enteral feeding was sufficient.

To distinguish scores 0 and 1, the mean value of the day was chosen. If the infant showed any sign of enterocolitis, the worst value of ‘2’ was selected. Furthermore, the mean value of hourly urine output between the last scoring time and the next scoring time was used to determine the score for the patient’s renal status. For creatinin value and the remaining systems, the worst value of the day was selected to determine the score for each system. Since the neurologic score was evaluated by cranial ultrasound for these two scoring systems, the score was calculated based on the last cranial ultrasound if it was not performed daily. If a value was missing for each score, it was assumed to be within the normal range. As the majority of infants are intubated and sedated during the first postoperative day, all patients sedated or under surgical anesthesia were considered to have a normal Glasgow coma scale score on day 1.

### 2.4. Statistical Analysis

Descriptive analysis was performed for the demographic and clinical characteristics of the infants. Categorical variables were described as percentages and compared using Pearson’s chi-squared test and Fisher’s exact test when necessary. For continuous variables, mean and standard deviation (SD) or medians with interquartile range (IQR) were given as descriptive variables. Normality of data was analyzed by using Kolmogorov–Smirnov test. Mann–Whitney U test was used for comparing PELOD-2, NEOMOD, and modified NEOMOD scores between survivors and non-survivors on day 1, day 2, and day 3.

Three main statistical analyses were used for comparing organ dysfunction scores to predict 30-day mortality. Hosmer–Lemeshow goodness-of-fit test was applied to assess calibration of each score on day 1, day 2, and day 3. A high *p*-value (>0.05) in this test indicated good calibration. The discriminative ability of PELOD-2, NEOMOD, and modified NEOMOD in predicting mortality were assessed by the area under the curve (AUC) of receiver operating characteristic (ROC) curves for each time point. In accordance with the literature, we considered AUC values of 0.9 or higher as excellent; 0.8 to 0.89 as very good; 0.7 to 0.79 as good; 0.6 to 0.69 as moderate; and <0.6 as poor [14].

Furthermore, we performed a pairwise comparison of the receiver operating characteristic curves of each mortality risk score for each time point using the DeLong method [15].

Youden index was also used to determine the best cut-off values. The higher index shows better prediction at the cut-off points [16]. Based on the optimal cutoff value, we calculated the sensitivity, specificity, positive predictive value (PPV), and negative predictive value (NPV) for assessment of both rapid scoring systems.

Statistical analyses were performed by using SPSS 23.0 (SPSS for Windows, version 23.0; SPSS, Inc., Chicago, IL, USA) and MedCalc Statistical Software version 19.2.6 (MedCalc Software bv, Ostend, Belgium; 2020).

## 3. Results

Of the initial cohort of 145 infants who met the inclusion criteria, 7 patients who died within the first 12 h after surgery were subsequently excluded from the study. The mean gestational age and birthweight for the entire cohort were 37.6 ± 3.5 weeks and 3104 ± 493 g, respectively. RACHS-1 score was ≥4 in 66 (47.8%) of the patients. The incidence of single ventricle anatomy, RACHS-1 score of ≥4, and delayed sternal closure were higher in the mortality group (*p* < 0.01, *p* = 0.02 and *p* < 0.01, respectively). Furthermore, the mortality group also had higher maximum VIS score on postoperative day 2 and higher incidence of acute kidney injury and necrotising enterocolitis in the postoperative period (*p* = 0.04, *p* < 0.01, and *p* = 0.03, respectively). The demographic and perioperative characteristics of the infants are summarized in Table 1.

Of the infants, 41 (29.7%) had single ventricle physiology. The most common underlying diagnosis was transposition of great arteries (TGA). Table 2 displays diagnoses of congenital heart disease of the entire cohort.

The total mortality rate of the entire cohort was 15.9%. The mortality distribution among infants over time was as follows: seven infants within the 12–24 h, five infants on day 2, five infants between day 2 and day 7, and five infants after the postoperative first week.

The median scores of PELOD-2, NEOMOD and modified NEOMOD was higher in the mortality group at each measurement point compared to the survivors (Table 3).

**Figure 1:**
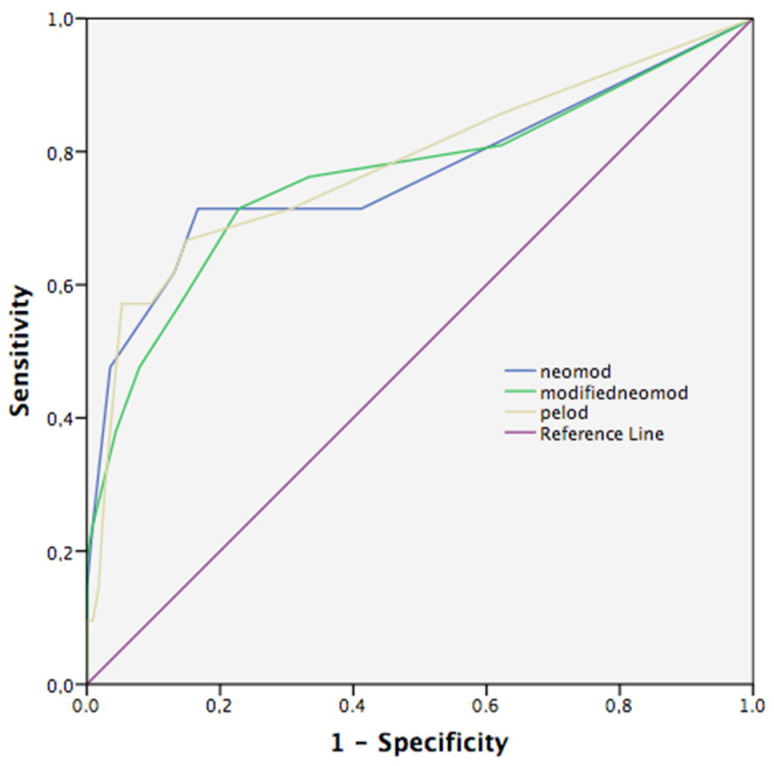
Receiver operating curves (ROC) of mortality on PELOD-2, NEOMOD, and modified NEOMOD on day 1. PELOD-2 score performed the best on day 1, but pairwise comparison did not show statistical difference between scores. (PELOD-2 vs. NEOMOD, *p* = 0.76, PELOD-2 vs. modified NEOMOD, *p* = 0.68, NEOMOD vs. modified NEOMOD, *p* = 0.92) PELOD-2, Pediatric Logistic Organ Dysfunction-2 score; NEOMOD, Neonatal Organ Dysfunction score.

The PELOD-2 score provided better prediction compared to the other two scores on day 1, while all three scoring systems predicted mortality statistically similar to each other (Figure 1).

**Figure 2:**
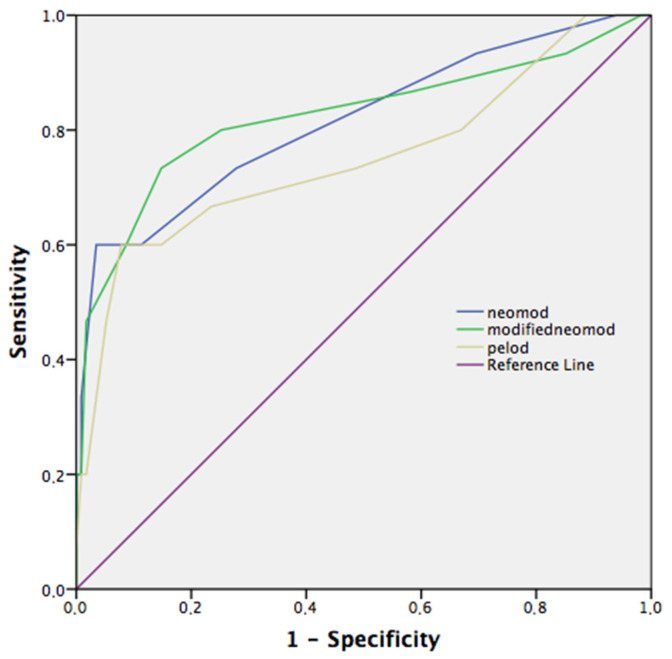
Receiver operating curves (ROC) of mortality on PELOD-2, NEOMOD, and modified NEOMOD on day 2. Modified NEOMOD score on day 2 performed the best overall, but pairwise comparison did not show statistical difference between modified NEOMOD and NEMOD, and PELOD-2 scores (*p* = 0.76, *p* = 0.27, respectively). PELOD-2, Pediatric Logistic Organ Dysfunction-2 score; NEOMOD, Neonatal Organ Dysfunction score.

The PELOD-2 demonstrated the best predictive ability on day 1, with the greatest AUC value (0.780, CI: 0.7–0.85) compared to day 2 and day 3 (0.750, CI: 0.67–0.82; 0.738, CI: 0.62–0.87, respectively). The modified NEOMOD score was more succesfull predicting mortality compared to the other two scores on day 2 (AUC: 0.824, CI: 0.75–0.89). However, no significant difference was found compared to NEOMOD and PELOD-2 on day 2 (*p* = 0.76, *p* = 0.27, respectively) (Figure 2).

**Figure 3:**
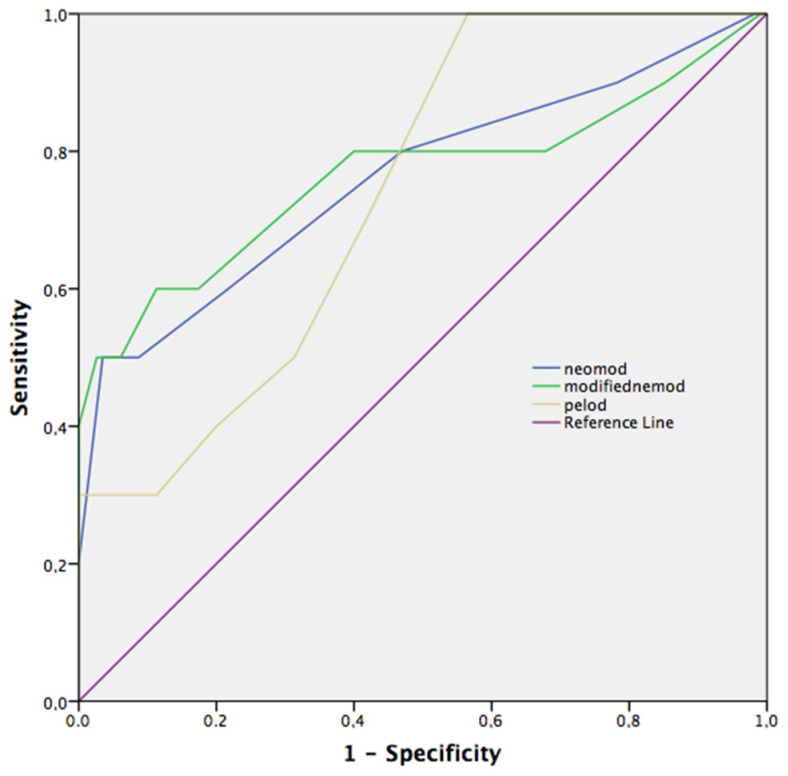
Receiver operating curves (ROC) of mortality on PELOD-2, NEOMOD, and modified NEOMOD on day 3. Modified NEOMOD score on day 3 performed the best overall, but pairwise comparison did not show statistical difference between modified NEOMOD and NEMOD, and PELOD-2 scores (*p* = 0.88, *p* = 0.71, respectively). PELOD-2, Pediatric Logistic Organ Dysfunction-2 score; NEOMOD, Neonatal Organ Dysfunction score.

Both NEOMOD and the modified NEOMOD scores had better AUC values than PELOD-2 on day 3, but pairwise comparison of ROC curves did not differ between scoring systems (NEOMOD vs. modified NEOMOD, *p* = 0.88, NEOMOD vs. PELOD-2, *p* = 0.71, modified NEOMOD vs. PELOD-2, *p* = 0.76) (Figure 3).

The Hosmer–Lemeshow goodness-of-fit test was not significant for every organ dysfunction score in each time periods, with all *p*  > 0.05 for χ^2^ ranging from 3.98 to 9.0, indicating good calibration (Table 4).

The modified NEOMOD on day 2 had the best overall performance with the highest Youden index of 0.59. It achieved a sensitivity of 73% and specificity of 85%. The remaining results are summarized in Table 5.

## 4. Discussion

In this retrospective study, we compared three organ dysfunction scores to predict 30-day mortality in neonates who underwent cardiac surgery. Our study indicates that both NEOMOD, modified NEOMOD, and PELOD-2 scores calculated in the first 3 postoperative days showed good discriminatory power with acceptable calibration in predicting mortality. Furthermore, the modified NEOMOD score on day 2 presented the best predictive ability with the greatest AUC value, classified as very good. To our knowledge, this is the first study that evaluates the performances of neonatal multiple organ dysfunction scores in predicting postoperative mortality in neonatal cardiac surgery patients.

Severity of illness scores play a crucial role in providing objective measures of critical illness and are valuable tools for enhancing the quality of care in intensive care units. Therefore, the absence of specific physiology-based severity of illness scores for neonates and older children who underwent cardiac surgery is a significant gap in current postoperative care. Currently available pediatric scoring systems that have been evaluated for assessing postoperative mortality in this population demonstrate variable predictive ability [17,18]. Moreover, the majority of these studies included neonates without clear stratification in terms of outcomes, and none of the pediatric scoring systems have been validated for a neonatal population. Studies have shown that there are significant differences in physiology and organ response to injury between neonates and older children [19,20]. These differences make neonates particularly susceptible to increased incidence of Low Cardiac Output Syndrome (LCOS), organ dysfunction, and consequently, mortality in the postoperative period after cardiac surgery [21]. This conclusion is further supported by Shime et al., who showed higher mortality related to organ dysfunction in neonates compared to the pediatric population in the postoperative period [5].

Consistent with these findings, our study showed organ dysfunction scores, PELOD-2, NEOMOD, and modified NEOMOD, demonstrated good prognostic ability for mortality in a neonatal cardiac population. However, we observed variations in the prognostic power of these scores during the initial three days. The discrimination of the PELOD-2 score was best on day 1 with an AUC value of 0.780, performing better than other two scores. Nevertheless, its predictive capability diminished over the subsequent days, reaching its lowest value on day 3, with an AUC of 0.738. This finding could potentially be attributed to the higher weight given to the cardiovascular score compared to other systems in the calculation of the total PELOD-2 score. However, not counting inotropic support, treatment in the regulation of mean arterial pressure may have resulted in reduced predictive ability in the following days. Furthermore, PELOD-2 uses the serum creatinin level as a single variable determining renal function. As recent studies showed delayed rise in the serum creatinin level and the role of the correction of serum creatinine for fluid balance in cardiac patients, it is possible that the PELOD-2 score may have underestimated the prevalence and impact of acute kidney injury in neonates [22].

The performance of NEOMOD and modified NEOMOD scores was consistently good, reaching their peak on day 2 with a very good ability to predict 30-day mortality. Both scores outperformed PELOD-2 after the initial postoperative day, but the statistical difference did not reach significance. However, both scores seem to have lower discrimination power when compared to the general population of premature infants and critically ill neonates, as indicated by excellent AUC values exceeding 0.900 in these specific subpopulations [9,10,23]. This disparity in performance could be due to the fact that these scores were initially designed to assess organ dysfunction in premature infants, with equal emphasis placed on each organ function included in the scoring system. Nonetheless, one of the primary factors contributing to mortality following cardiac surgery is expected to be the impact of myocardial edema and ischemia-reperfusion injury on ventricular performance, coupled with the limited capacity of the immature neonatal heart to enhance ventricular function [24]. To potentially improve the predictive ability of these scores in neonatal cardiac populations, modifications could be made by assigning greater weight to the cardiovascular component. Furthermore, it can be suggested that these two scores may perform better in premature infants undergoing cardiac surgery.

Previous studies have shown microvascular system involvement characterized by edema and unexplained persistent weight gain might be the earliest signs of MODS in term surgical neonates [19,25]. Additionally, fluid overload and lower albumin levels in the perioperative period have been independently associated with worse outcomes in the neonatal cardiac population [26,27]. These findings led us to hypothesize that the modified NEOMOD score, which includes a component assessing microvascular dysfunction (degree of edema, albumin), would have the best performance in predicting mortality. Our results might support this hypothesis, as the modified NEOMOD score consistently demonstrated the highest AUC values after the first day among the three scores evaluated. However, the difference in performance between NEOMOD and modified NEOMOD scores was not statistically significant on each day. We speculate that this lack of significance may be caused by the use of intraoperative and early postoperative albumin infusions for reasons other than hypoalbunemia and the prophylactic peritoneal dialysis treatment in some patients.

One of the major challenges in using these scores in our study was obtaining a neurologic dysfunction score. Both NEOMOD and modified NEOMOD scores require cranial ultrasound assessment to evaluate neurologic dysfunction, as their classification is based on preterm brain injury and they do not count altered conscious states. In addition, the Glasgow Coma Scale, the neurologic component of PELOD-2 score, was difficult to interpret in neonates due to their neurological immaturity. Although the neurological variables of all patients were evaluated by the same physicians, the possibility of incorrect interpretation of neurologic dysfunction in some patients could potentially diminish the predictive performance of these scores in the recent study. Given that the primary objective of our study was to compare the predictive ability of the scores in their current form for mortality, we did not modify the neurological variables. Furthermore, our observation was in line with previous studies that it was not feasible to interpret neurologic scores when the patients were under sedation [5,28]. Future research should address the use of appropriate neurological scores in sedated infants after surgery.

This study is subject to certain inherent limitations. First, it is a retrospective study in a single unit with a relatively small sample size. Second, the scarcity of the literature on the term neonatal MODS, especially in surgical neonates, challenged us to determine the optimal time points for score calculation and not enough data were available to allow the daily calculation of all scores for an extended period. Third, although neonatal organ failure scores were used for the estimation of outcomes in critically ill term neonates, a validated organ dysfunction score in term neonatal population is lacking.

In conclusion, our study is the first ever to study organ dysfunction scores in mortality prediction in neonatal cardiac surgery. The modified NEOMOD score showed the best predictive ability after the initial 24 h following surgery, with its peak performance observed on day 2. Further studies are required to improve our understanding of the MODS in neonatal cardiac patients and validate the efficacy of these organ dysfunction scores for this specific subpopulation.

## Figures and Tables

**Table 1:** Baseline characteristics, preoperative, perioperative, and postoperative data of the study group.

	Total Cohort (*n* = 138)	Survivors (*n* = 116)	Non-Survivors (*n* = 22)	*p* Value *
*Infant Characteristics*				
Gestational age, (weeks) (SD)	37.6 ± 3.5	37.5 ± 3.39	38 ± 1.09	0.88
Gender (male), *n* (%)	79 (57.2)	70 (60.3)	9 (40.9)	0.07
Birth weight, (grams) (SD)	3104 ± 493	3107 ± 491	3088 ± 516	0.59
Premature, *n* (*%*)	15 (10.9)	13 (11.2)	2 (9)	0.76
SGA, *n* (*%*)	12 (8.7)	9 (7.8)	3 (13.6)	0.41
Weight at surgery, (grams) (SD)	3267 ± 454	3276 ± 458	3215 ± 435	0.46
Age at surgery, d, median (IQR)	13 (9–19)	13 (9–19.5)	11.5 (6–19)	0.28
Cardiovascular diagnosis, *n (%)*				
Single ventricle anatomy	41 (29.7)	27 (23.3)	14 (63.6)	<0.01
Double ventricular anatomy	97 (70.3)	89 (76.7)	8 (36.4)	
RACHS-1 score, *n (%)*				
<4	72 (52.2)	66 (56.9)	6 (27.3)	0.02
≥4	66 (47.8)	50 (43.1)	16 (72.7)	
Preoperative mechanical ventilation, *n* (%)	98 (71)	80 (68.9)	18 (81.8)	0.3
Preoperative organ failure, *n* (%)	16 (11.6)	13 (11.2)	3 (13.6)	0.72
*Perioperative Data*				
Cardiopulmonary bypass (min), median (IQR)	182 (125–211)	182 (132–210)	180.5 (117–220)	0.69
Aortic cross clamp (min), median (IQR)	120 (76–146)	121 (90–147)	102 (53–146)	0.14
Delayed sternal closure, *n* (%)	37 (26.8)	24 (20.7)	13 (59.1)	<0.01
*Postoperative Data*				
Maximum VIS, day 1, median (IQR)	25 (16.5–35)	23 (15–35)	25 (20–40.25)	0.25
Maximum VIS, day 2, median (IQR)	15 (15–26)	13 (8–20)	31 (16.25–45.5)	0.04
Postoperative complications				
AKI, *n* (%)	54 (39.1)	40 (34.4)	14 (63.6)	<0.01
Peritoneal dialysis, *n* (%)	22 (15.9)	10 (8.6)	12 (54.5)	<0.01
Necrotizing enterocolitis, *n* (%)	11 (8)	8 (6.9)	3 (13.6)	0.03
Chylothorax, *n* (%)	3 (2.2)	3 (2.6)	-	1
Sepsis, *n* (%)	33 (23.9)	29 (25)	4 (18.2)	0.48
Arrythmia, *n* (%)	22 (15.9)	19 (16.3)	2 (9.1)	0.62
Neurologic dysfunction, *n* (%)	8 (5.8)	6 (5.2)	2 (9)	0.21
*Postoperative*				
NICU length of stay, day, median (IQR)		13 (8–20)	2 (1–7.75)	<0.01
Hospital length of stay, day, median (IQR)		18.5 (13–27)	2 (1–7.75)	<0.01

SGA, small for gestational age; RACHS-1, Risk Adjustment for Congenital Heart Surgery; VIS, Vasoactive inotropic score; NICU, neonatal intensive care unit. Plus–minus values are mean ± standard deviation SD: standard deviation. IQR: interquartile range * p values < 0.05 were considered as significant.

**Table 2:** Type of Congenital Heart Disease of the neonates who underwent cardiac surgery.

Underlying Diagnosis, *n*	Total Cohort (*n* = 138)
TGA/TGA-VSD	68
Aortic Arch Hypoplasia/left ventricular hypoplasia	3
HLHS/HLHS variants	25
Pulmonary atresia	8
TAPVR	4
DORV-remote VSD/single ventricle	3
Aortic interruption	5
Taussig–Bing anomaly	2
Tricuspid atresia	4
Complex univentricular heart disease	10
Unbalanced complete AVSD/aortic atresia	2
Truncus arteriosus	2
Tetralogy of fallot/pulmonary atresia	2

TGA, Transposition of great arteries; HLHS, Hypoplastic left heart syndrome; TAPVR, Total anomalous pulmonary venous return; DOVR, Double outlet right ventricle; VSD, ventricular septal defect; AVSD, atrioventricular septal defect.

**Table 3:** Comparison of PELOD-2, NEOMOD, and modified NEOMOD scores in survivors and non-survivors.

	Survivors	Non-Survivors	*p* Value *
**Day 1 (*n* = 138), median (IQR)**			
PELOD-2 score	5 (4–6)	10 (5–11)	<0.01
NEOMOD score	4 (4–5)	7 (4–8.25)	<0.01
Modified NEOMOD score	5 (4–6)	8 (5.75–10.25)	<0.01
**Day 2 (*n* = 131), median (IQR)**			
PELOD-2 score	5 (4–6)	9 (5.25–11)	<0.01
NEOMOD score	4 (3–5)	7 (4.25–8)	<0.01
Modified NEOMOD score	5 (4–5.75)	8 (6.25–9)	<0.01
**Day 3 (*n* = 126), median (IQR)**			
PELOD-2 score	4 (3–6)	7.5 (5–14)	<0.01
NEOMOD score	3 (3–4)	6 (4–8)	<0.01
Modified NEOMOD score	4 (3–5)	8 (5–12)	<0.01

PELOD-2, Pediatric Logistic Organ Dysfunction-2 score; NEOMOD, Neonatal Organ Dysfunction score. IQR: interquartile range * p values < 0.05 were considered as significant.

**Table 4:** Comparison of organ dysfunction scores in predicting mortality according to study days.

	Discriminatory Ability ^a^		Calibration Using Hosmer–Lemeshow	
			Goodness-of-Fit Test ^b^	
	AUC	95%CI	χ^2^	*p* Value
**Day 1**				
PELOD-2 score	0.78	(0.70–0.85)	3.98	0.55
NEOMOD score	0.766	(0.69–0.84)	5.13	0.08
Modified NEOMOD score	0.763	(0.68–0.83)	6.23	0.4
**Day 2**				
PELOD-2 score	0.75	(0.67–0.82)	7.2	0.41
NEOMOD score	0.815	(0.74–0.88)	4.8	0.57
Modified NEOMOD score	0.824	(0.75–0.89)	4.69	0.7
**Day 3**				
PELOD-2 score	0.738	(0.62–0.87)	8.66	0.28
NEOMOD score	0.753	(0.67–0.84)	8.17	0.32
Modified NEOMOD score	0.764	(0.68–0.85)	9	0.19

PELOD-2, Pediatric Logistic Organ Dysfunction-2 score; NEOMOD, Neonatal Organ Dysfunction score. ^a^ There is no difference between groups when groups are compared with each other on each day (*p* > 0.05). ^b^ The null hypothesis of the Hosmer–Lemeshow goodness-of-fit test is that the assessed score predicts death correctly. Thus, a significant *p* value indicates poor calibration.

**Table 5:** Measure of performances of organ dysfunction scores in predicting mortality according to study days.

	% (95%CI)					
	Cut-Off	Youden	Sensitivity	Specificity	PPV (%)	NPV (%)
	Value	Index	(%)	(%)		
**Day 1**						
PELOD-2 score	>9	0.52	57 (34–78)	95 (89–98)	67 (46–83)	92 (88–95)
NEOMOD score	>5	0.54	71 (48–89)	83 (74–89)	43 (32–55)	94 (89–97)
Modified NEOMOD score	>6	0.51	71 (48–89)	77 (68–85)	37 (27–47)	94 (88–97)
**Day 2**						
PELOD-2 score	>8	0.52	60 (32–84)	92 (86–96)	50 (32–68)	95 (91–97)
NEOMOD score	>6	0.55	60 (32–97)	97 (91–99)	69 (44–87)	95 (91–97)
Modified NEOMOD score	>6	0.59	73 (45–92)	85 (77–91)	39 (28–53)	96 (92–98)
**Day 3**						
PELOD-2 score	>3	0.43	70 (35–93)	58 (49–67)	13 (8–29)	96 (90–98)
NEOMOD score	>6	0.47	50 (18–81)	97 (91–99)	56 (29–78)	96 (92–98)
Modified NEOMOD score	>6	0.49	60 (26–88)	89 (81–94)	32 (18–49)	96 (92–98)

PELOD-2, Pediatric Logistic Organ Dysfunction-2 score; NEOMOD, Neonatal Organ Dysfunction Score.

## Data Availability

The authors confirm that the data supporting the findings of this study are available within the article.
